# Modeling of lamin A/C mutation premature cardiac aging using patient-specific induced pluripotent stem cells

**DOI:** 10.18632/aging.100503

**Published:** 2012-12-03

**Authors:** Chung-Wah Siu, Yee-Ki Lee, Jenny Chung-Yee Ho, Wing-Hon Lai, Yau-Chi Chan, Kwong-Man Ng, Lai-Yung Wong, Ka-Wing Au, Yee-Man Lau, Jinqiu Zhang, Kenneth Weijian Lay, Alan Colman, Hung-Fat Tse

**Affiliations:** ^1^ Cardiology Division, Department of Medicine, Queen Mary Hospital, the University of Hong Kong, Hong Kong SAR, China; ^2^ Research Center of Heart, Brain, Hormone and Healthy Aging, Li Ka Shing Faculty of Medicine, the University of Hong Kong, Hong Kong SAR, China; ^3^ Institute of Medical Biology, A*STAR Institute of Medical Biology, Singapore

**Keywords:** Dilated cardiomyopathy, induced pluripotent stem cells, LMNA

## Abstract

**AIMS:**

We identified an autosomal dominant non-sense mutation (R225X) in exon 4 of the lamin *A/C (LMNA)* gene in a Chinese family spanning 3 generations with familial dilated cardiomyopathy (DCM). In present study, we aim to generate induced pluripotent stem cells derived cardiomyocytes (iPSC-CMs) from an affected patient with R225X and another patient bearing *LMNA* frame-shift mutation for drug screening.

**METHODS and RESULTS:**

Higher prevalence of nuclear bleb formation and micronucleation was present in *LMNA^R225X/WT^ and LMNA^Framshift/WT^* iPSC-CMs. Under field electrical stimulation, percentage of LMNA-mutated iPSC-CMs exhibiting nuclear senescence and cellular apoptosis markedly increased. shRNA knockdown of LMNA replicated those phenotypes of the mutated LMNA field electrical stress. Pharmacological blockade of ERK1/2 pathway with MEK1/2 inhibitors, U0126 and selumetinib (AZD6244) significantly attenuated the pro-apoptotic effects of field electric stimulation on the mutated *LMNA* iPSC-CMs.

**CONCLUSION:**

LMNA-related DCM was modeled in-vitro using patient-specific iPSC-CMs. Our results demonstrated that haploinsufficiency due to R225X *LMNA* non-sense mutation was associated with accelerated nuclear senescence and apoptosis of iPSC- CMs under electrical stimulation, which can be significantly attenuated by therapeutic blockade of stress-related ERK1/2 pathway.

## INTRODUCTION

Lamins A and C are intermediate filament proteins encoded by lamin A/C gene (*LMNA*) and constitute major components of nuclear lamina [1]. Mutations in *LMNA* have been shown to cause a wide range of human diseases collectively referred to as “laminopathies,”[2-6] from Hutchinson Gilford Progeria (premature aging syndrome), muscular dystrophy, to familial dilated cardiomyopathy (DCM). *LMNA-*related DCM is characterized by early onset of atrial fibrillation and conduction system disease, and subsequent progression to sudden cardiac death and heart failure [7, 8]. Indeed, *LMNA* mutations are the most common cause of familial DCM, accounting for 5-10% of overall familial DCM and up to 30-45% families with DCM and conduction system disease [9, 10]. Although the age of presentation in *LMNA-*related DCM can range from the first to sixth decade of life, almost all patients become symptomatic after age 60 [7, 11, 12]. Furthermore, *LMNA-*related DCM, especially in those associated with conductive system disease have a more malignant clinical course than other familial DCM due to a high rates of progressive heart failure and sudden cardiac death due to ventricular tachyarrhythmias [12-15]. Despite our increasing awareness on the importance of *LMNA-*relatedDCM, the mechanism of the disease as well as the therapeutic strategies to prevent the onset and progression of disease remain unclear.

Several animal models of *LMNA* mutations have been generated to provide initial insights into the pathophysiology for *LMNA*-related DCM[16-18]. In these animal models, either knock-in of mutant *LMNA* (dominant negative)[17] or knock-out of *LMNA* (haploinsufficieny) cause DCM in mice [16, 18], but variable cardiac phenotypes with or without conduction system disease were observed. The high mortality of the knockout mice also restricts the possibility of the whole animal study. As a result, the mechanisms by which different *LMNA* mutations cause DCM remain uncertain. An *in-vitro* platform of human cardiomyocytes derived from patients with different *LMNA* mutations would be extremely useful for understanding disease mechanism and for testing patient-specific therapies.

Latest breakthrough in the generation of human induced pluripotent stem cells (iPSC) from adult somatic tissues [19, 20] provides a unique opportunity to produce patient-specific cardiomyocytes for disease modeling and drug screening[21-23]. We and others [24, 25] have recently reported the use of human iPSC platform to model the disease phenotypes and mechanisms of Hutchinson-Gilford progeria syndrome, which is the most severe form of *LMNA* mutation that leads to premature aging and death. Here, we propose to recapitulate the disease phenotype, pathophysiology and drug screening for *LMNA*-related familial DCM *in-vitro* using cardiomyocytes derived from patient-specific human iPSCs (iPSC-CMs).

## RESULTS

### Altered nuclear architecture and enhanced electric-stimulation induced apoptosis in LMNA^R225X/WT^ dermal fibroblasts

**Figure 1:**
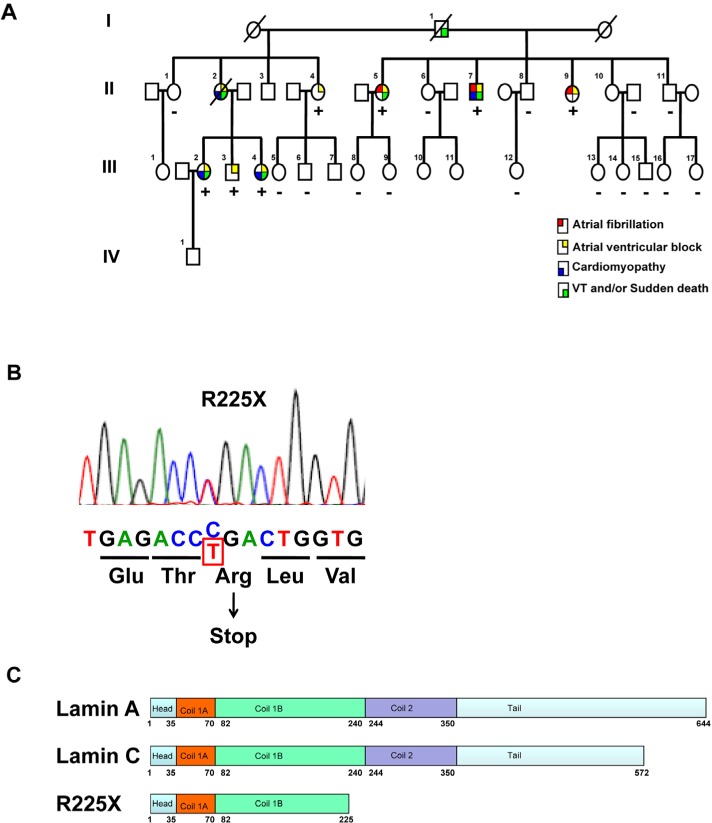
Clinical phenotype and *LMNA* genotype **(A)** The pedigree of the proband (Patient II-7) with cardiac laminopathy featuring atrial fibrillation, atrioventricular block, dilated cardiomyopathy, ventricular tachyarrhythmia and sudden cardiac death. Four-generation family had 9 affected members showing autosomal-dominant cardiac disease. (See also table 1)(Square = male; circle = female; slash = deceased). A nonsense *LMNA* mutation (R225X) segregates with atrial fibrillation, atrioventricular block, dilated cardiomyopathy and ventricular tachyarrhythmia (“+” = *LMNA*^R225X/WT^, “-” = *LMNA*^WT/WT^). **(B)** The sequence chromatogram of the mutant allele of *LMNA*. The mutation altering amino acid sequence (in red) predicts a premature stop codon. **(C)** The schematic of lamin A/C proteins. Alternative splicing results at 572 and 664 amino acid lamin C and lamin A proteins. The R225X mutation results in a premature stop codon in the α-helical rod domain, thus a truncated lamin protein.

**Figure 2:**
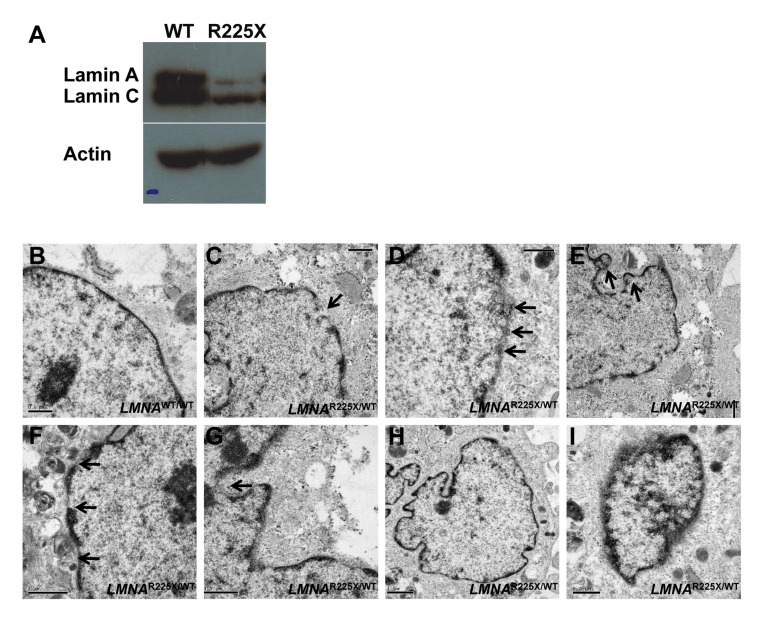
*LMNA*^R225X/WT^ dermal fibroblasts showing nuclear defects and accelerated apoptosis upon electrical stimulation **(A)** The expressions of lamin A/C proteins in control and *LMNA*^R225X/WT^ dermal fibroblasts with western blot analysis using anti-*LMNA* antibody targeting both N-terminus. **(B-I)** Electronic micrographs of nuclei of cultured dermal fibroblasts from controls and *LMNA*^R225X/WT^: **(B)** Normal nuclear envelope in control dermal fibroblast;**(C)** Focal loss of the nuclear membrane in *LMNA*^R225X/WT^ dermal fibroblasts (arrow);**(D)** Clustering of nuclear pores;**(E, F & G)** Bleb and micronucleus formation; **(H)** Accumulation of mitochondria around the nuclear envelope; **(H & I)** Irregular shape nucleus, and condensed chromatin.

**Figure 3:**
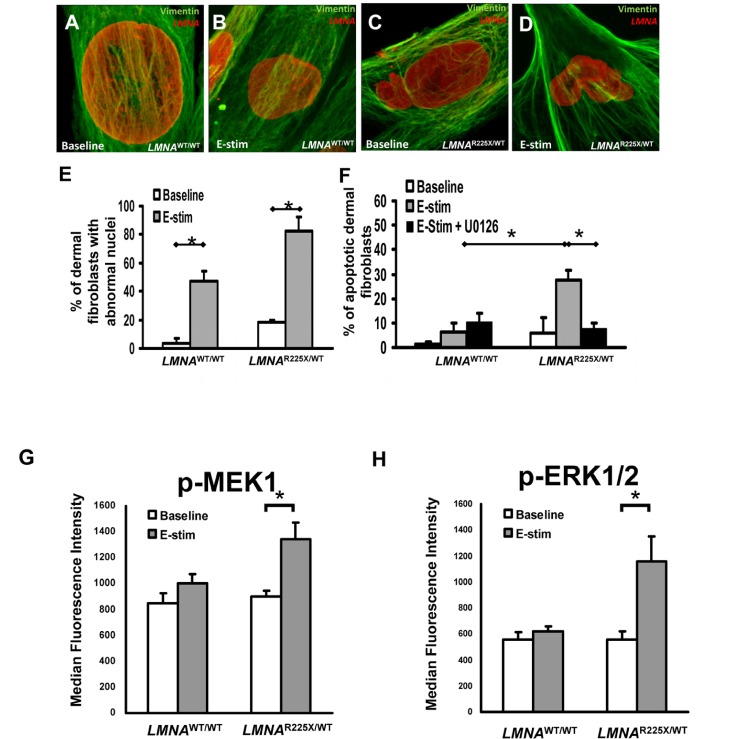
Effect of electrical stimulation on *LMNA*^R225X/WT^ dermal fibroblasts **(A-D)** Immunofluorescence showing typical nuclear morphologies of control and *LMNA*^R225X/WT^ dermal fibroblasts before and after electrical stimulation. **(E)** Effects of electrical stimulation on the percentage of control and *LMNA*^R225X/WT^ dermal fibroblasts with nuclear blebs. **(F)** Effects of electrical stimulation and U0126 (ERK1/2 pathway blocker) on the apoptosis of control and *LMNA*^R225X/WT^ dermal fibroblasts. The application of U0126, a highly selective blocker of the MEK1 pathway, nearly completely abolished the apoptotic effects of electrical stimulation on *LMNA*^R225X/WT^ dermal fibroblasts. **(G-H)** Electrical stimulation-activates mitogen-activated protein kinases (MAPK) pathway in iPSC-CM: **(G)** MEK1 and **(H)** extracellular signal-regulated protein kinases 1 and 2 (ERK1/2) in *LMNA*^R225X/WT^ dermal fibroblasts, but not in control fibroblasts. *p- value <0.05.

We have established primary cultures of dermal fibroblasts from the proband (II-7) harboring the heterozygous R225X *LMNA* mutation and from a control individual (Figure 1). Compared with control dermal fibroblasts, *LMNA*^R225X/WT^ dermal fibroblasts had reduced expression of the lamin A/C proteins (Figure 2A). Ultra-structural analysis using electronic microscopy revealed dramatic morphological alterations suggestive of nuclear senescence in *LMNA*^R225X/WT^ dermal fibroblasts including focal loss of nuclear membrane, clustering of nuclear pores, large aggregates of highly condensed heterochromatin, bleb and micronucleus formation, and accumulation of mitochondria around the nuclear envelope (Figure 2B-I). Although these dysmorphic features of nuclear senescence, commonly seen in aging cells [26], could also be observed in the control fibroblasts with immunofluorescence analysis, they occurred in lower frequency (3.6 ± 3.6%, *vs.* 18.0 ± 1.8%, *P<0.05*) with less-severe morphological abnormalities in comparison with *LMNA*^R225X/WT^ dermal fibroblasts (Figure 3A-E).

Previous study in a lamin A-haploinsufficiency mouse model of DCM had suggested that reduction of lamin A/C proteins render premature apoptosis, particularly in electrically active cardiomyocytes [18.] Therefore, we applied electrical stimulation to cultured dermal fibroblasts to simulate an *in-vivo* cardiac electric field, and scored the fractions of abnormal nuclei present in control and *LMNA*^R225X/WT^ dermal fibroblasts (Figure 3A-D). After electrical stimulation (at 6.6 V/cm, 1 Hz for 3 days), the percentage of cell with nuclear senescence (82 ± 9.7% *vs.* 47.4 ± 7.1%, *P<0.05*) and apoptosis (27.5 ± 4.1% *vs*. 6.5 ± 3.4%, *P<0.05*) were significantly higher in *LMNA*^R225X/WT^ dermal fibroblasts as compared with control dermal fibroblasts (Figure 3E-F).

### Mechanisms of electrical stimulation-induced apoptosis

We further explored the possible stress-related signaling pathway involved in electrical stimulation-induced apoptosis in *LMNA*^R225X/WT^ dermal fibroblasts. Upon electrical stimulation, MEK1 and its downstream candidate, extracellular signal-regulated protein kinases 1 and 2 (ERK1/2), were activated only in *LMNA*^R225X/WT^ dermal fibroblasts, but not in control fibroblasts (Figure 3G-H). The application of U0126, a highly selective blocker of the MEK1-ERK1/2 pathway, nearly completely abolished the apoptotic effects of electrical stimulation on *LMNA*^R225X/WT^ dermal fibroblasts (Figure 3F). These data suggest that increased nuclear senescence and apoptosis induced by electrical stimulation in *LMNA*^R225X/WT^ dermal fibroblasts was mediated via the MEK1 pathway.

### Generation of LMNA^R225X/WT^ iPSC and cardiac differentiation

Despite the near-ubiquitous *LMNA* expression in most somatic cells, DCM is the most prominent phenotype of the R225X mutation. Therefore, we generated iPSCs from dermal fibroblasts isolated from the proband (II-7) to derive cardiomyocytes for recapitulating the cardiac phenotype of *LMNA*-related familial DCM *in-vitro*.

Altogether 4 clones of iPSCs were generated from the proband; however, chromosomal translocation was detected in 1 of these clones. As a result, the subsequent experiments were from the remaining 3 clones, which we did not detect any significant clone-to-clone variation. The genomic *LMNA* was sequenced in all iPSCs, and the expected mutation, R225X in *LMNA* was detected from the iPSCs generated from the dermal fibroblasts of the proband but not from those of the control. Besides, as we previously generated another iPSC line derived from DCM patient bearing frame-shift mutated *LMNA [27]*, it was used as another *LMNA* halploinsuffiency model in the present study.

**Figure 4:**
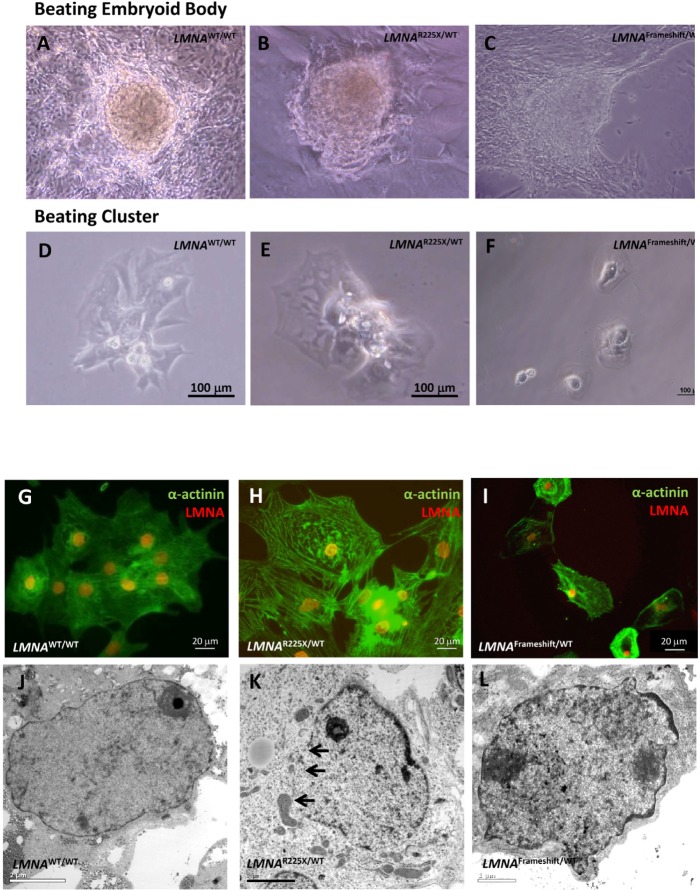
Cardiomyocytes derived from *LMNA*^R225X/WT^, *LMNA*^Frameshift/WT^ and *LMNA*^WT/WT^ iPSCs **(A, B & C)** Beating embryoid bodies derived from *LMNA*^R225X/WT^, *LMNA^Frameshift/WT^* and *LMNA*^WT/WT^ iPSCs;**(D, E & F)** After micro-surgical dissection, spontaneously beating cell clusters were plated onto glass coverslips. Videos of these beating embryoid bodies and clusters were available in supplemental materials. **(G, H & I)** Immunofluorescence co-staining showing the expression of cardiac specific marker, α-actinin and lamin A/C in these cells, **(J, K & L)** Electronic microscopy of nucleus of cardiomyocytes derived from *LMNA*^R225X/WT^, *LMNA^Frameshift/WT^* and *LMNA*^WT/WT^ iPSC line. The black arrows indicate the loss of nuclear envelope in the *LMNA*^R225X/WT^ iPSC-derived cardiomyocytes.

After cardiac differentiation, spontaneously beating outgrowths appeared approximately 14 to 21 days in embryoid bodies from both *LMNA*^R225X/WT^, *LMNA^Frameshift/WT^* and control iPSC lines (Figure 4A-C and *Supplementary video I-III*). The beating outgrowths were micro-surgically dissected out from the EBs and were dissociated into single cell clusters (Figure 4D-F, and *Supplementary video IV-VI*). Immunofluorescence analysis revealed the typical pattern of a cardiac-specific protein, α-actinin in beating cells from both *LMNA*^R225X/WT^, *LMNA^Frameshift/WT^* and control iPSC lines, confirming their cardiac identity (Figure 4G-I). Morphologically, there were no observable difference between the cardiomyocytes derived from the three iPSC lines, except the more prominent nuclear alterations of nuclei in *LMNA*^R225X/WT^ and*LMNA^Frameshift/WT^* iPSC-CMs (Figure 4J-L).

Furthermore, we characterized the electrophysiological properties of *LMNA*^R225X/WT^ iPSC-CMs using whole-cell patch clamp experiments. Figure 4I depicts representative single action potentials recorded from cardiomyocytes derived from *LMNA*^R225X/WT^ and control iPSCs. According to action potential recording, cells were then classified into atrial- and ventricular-like cardiomyocytes. There were no significant differences in the proportion of atrial-like and ventricular-like cardiomyocytes between *LMNA*^R225X/WT^ iPSCs and control. Furthermore, there were no significant differences in action potential duration at 50% and 90% repolarization (APD90) between the two groups (Data not shown).

### Susceptibility of iPSC-CMs to electrical stimulation

**Figure 5:**
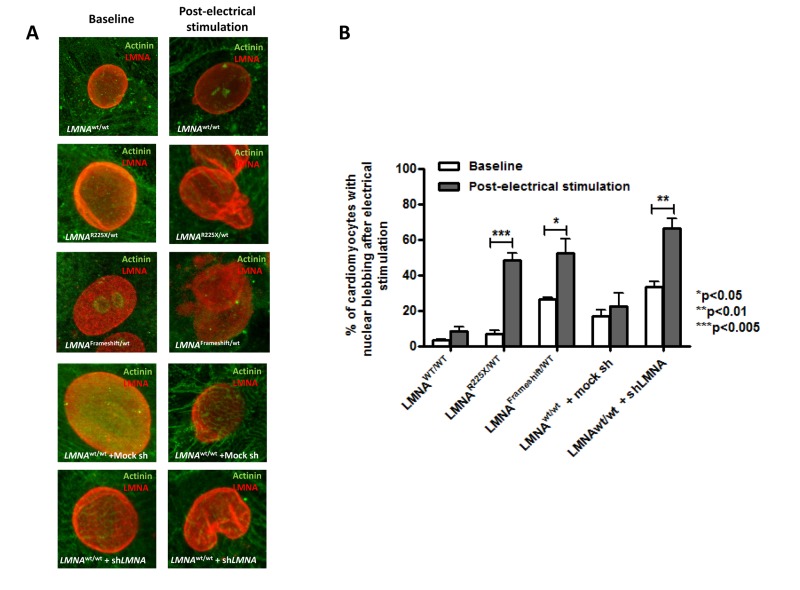
Electrical stimulation inducing nuclear abnormality in cardiomyocytes derived from *LMNA*^R225X/WT^ &*LMNA*^Frameshift/WT^ iPSCs **(A)** Representative immunofluorescence staining of cardiomyocytes derived from *LMNA*^WT/WT^ iPSCs, *LMNA*^R225X/WT^ iPSCs, *LMNA*^Frameshift/WT^ iPSCs, *LMNA*^WT/WT^ iPSCs treated with mock shRNA, and *LMNA*^WT/WT^ iPSCs treated with sh*LMNA* at baseline and after electrical stimulation. **(B)** The percentage of cardiomyocytes showed nuclear blebs.

**Figure 6:**
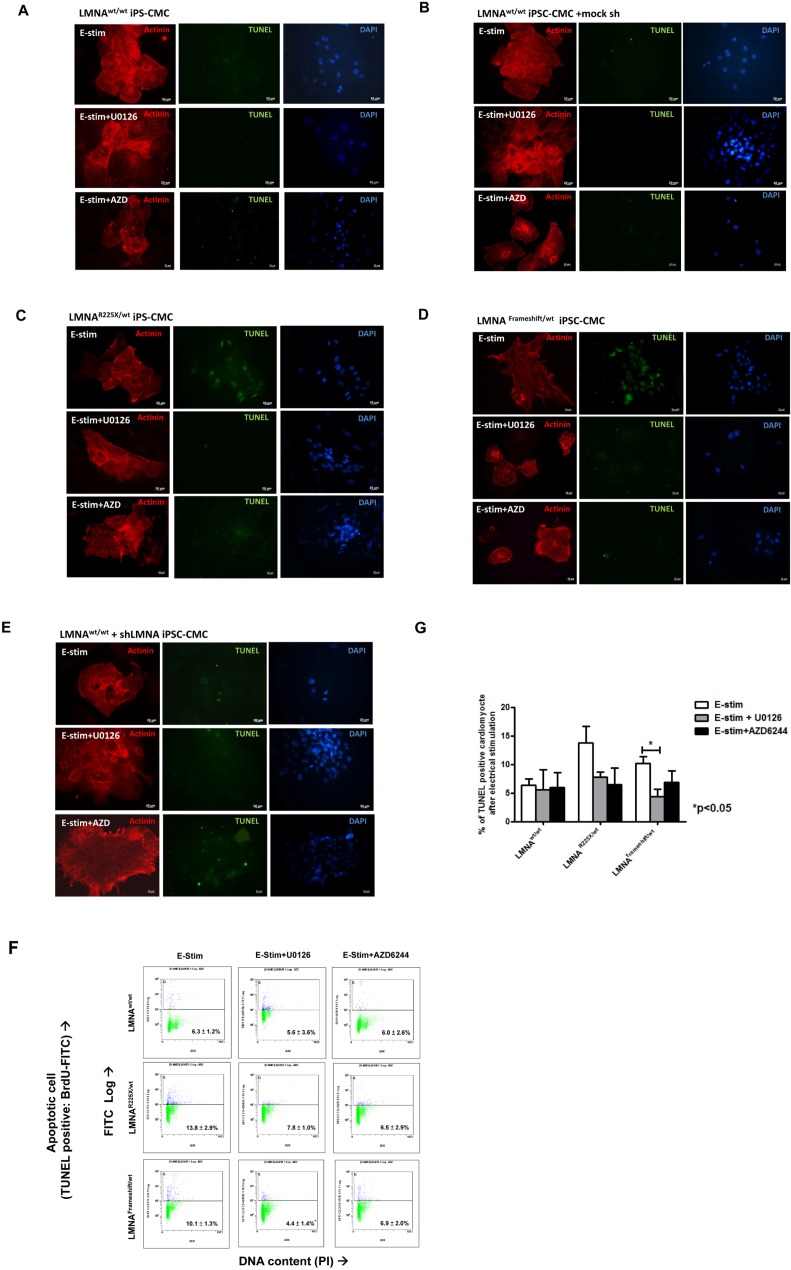
Electrical stimulation inducing apoptosis in cardiomyocytes derived from *LMNA*^R225X/WT^ &*LMNA*^Frameshift/WT^ iPSCs Representative TUNEL assay and co-immunofluorescence staining of alpha-actinin in cardiomyocytes derived from **(A)** *LMNA*^WT/WT^ iPSCs. **(B)** *LMNA*^R225X/WT^ iPSCs. **(C)** *LMNA*^Frameshift/WT^ iPSCs. **(D)** *LMNA*^WT/WT^ iPSCs treated with mock shRNA. **(E)** *LMNA*^WT/WT^ iPSCs treated with sh*LMNA* at baseline and after electrical stimulation. **(F, G)** Quantification of apoptotic cardiac differentiated iPSCs by APO-BrdU TUNEL assay. The percentage of cardiomyocytes with apoptosis was determined by FACS analysis by FL-1 positive gating. Unpaired t-test was performed between treatment and baseline n=3-5, *p-value<0.05.

Because electrical stimulation increased the fraction of cells with nuclear senescence as well as apoptosis in *LMNA*^R225X/WT^ dermal fibroblasts, we further tested whether *LMNA*^R225X/WT^ iPSC-CMs exhibited similar susceptibility to electrical stimulation. At baseline, there was no significant difference in the percentage of cardiomyocytes with nuclear senescence between the control and *LMNA*^R225X/WT^ iPSC-CMs (3.1 ± 0.7 % vs. 6.8 ± 2.2%) (Figure 5A-B). Upon electrical stimulation, the percentage of nuclear senescence in *LMNA*^R225X/WT^ and *LMNA*^Frameshift/WT^ iPSC-CMs markedly increased to 48.2 ±4.0% (*P<0.005* vs. baseline) and 52.3 ± 7.9% (*P<0.05* vs. baseline) respectively and was significantly higher than those of the control iPSC-CMs (8.6±2.5%, *P<0.01* in both mutated *LMNA* group; Figure 5B). More importantly, electrical stimulation resulted in a remarkably higher percentage of apoptotic cells in both *LMNA*^R225X/WT^ and *LMNA^Framshift/WT^* iPSC-CMs compared with control (13.8±2.9% and 11.2±0.8% vs. 6.3±1.2%; n=3 *P<0.05*) as determined by APO-BrdU TUNEL assay quantitatively (Figure 6F and G). Co-staining of cardiac marker with TUNEL reaction further qualitatively verify the apoptotic events happened in *LMNA*^R225X/WT^ and *LMNA^Framshift/WT^* iPSC-CMs upon electrical field stimulation.

To further verify whether lamin insufficiency contributes to the enhanced susceptibility of cardio-myocytes to electrical stimulation, we altered the expression of *LMNA* in control hiPSC-CMs by shRNA-mediated down-regulation (~60% as revealed by western blotting by probing of N-terminal LMNA A/C antibodies, data not shown). shRNA-treated control iPSC-CMs exhibited significantly higher percentage of nuclear senescence at baseline and after electrical stimulation (p<0.05) (Figure 5B). Likewise, shRNA mediated down-regulation of *LMNA* resulted in a significantly higher percentage of apoptotic cells than in control iPSC-CMs after electric stimulation (Figure 6E).

Intriguingly, the application of U0126 and selumetinib, AZD6244 (an anti-cancer drug in phase I-II clinical trial) to block MEK1 pathway in *LMNA*^R225X/WT^ and *LMNA^Framshift/WT^* iPSC-CMs and shRNA treated control iPSC-CMs showed a trend that attenuated or even completely abolished the apoptotic effects of field electric stimulation on these lamin-deficient cardiomyocytes as seen in the dermal fibroblast (Figure 3 and 6) whereas 10 μM of U0126 significantly rescued the rate of apoptosis mediated by electrical stress (11.2 ± 0.8% vs. 4.4 ± 1.4%; n=3, *P<0.05*). On the other hand, although it has recently demonstrated that rapamycin reverses progeric phenotype in laminopathy, the addition of rapamycin (0.68 mM) did not alleviate the electrical stimulation mediated apoptosis of mutant *LMNA* iPSC-CMs (Supplementary Figure 3).

## DISCUSSION

*LMNA*-related DCM is the most common form of familial DCM encountered in clinical practice. Affected individuals are often asymptomatic at early stage except with typical ECG findings of low amplitude P waves, prolonged PR intervals, but relatively normal QRS complexes. As the disease progresses with age, patients develop atrial fibrillation, progressive conduction block and left ventricular dysfunction, and sudden cardiac death due to life-threatening ventricular tachyarrhythmias [7, 12, 15]. Since the first description of isolated DCM related to *LMNA* mutations by Fatkin *et al* in 1999[7], there are more than 40 *LMNA* mutations related to familial DCM have been reported. Despite of numerous clinical reports, the pathogenic mechanisms by which a defect in nuclear envelope link to the clinical DCM remain elusive.

In this study, we first demonstrated that primary dermal fibroblast from patients with nonsense *LMNA*^R225X/WT^ mutation had reduced expression of the lamin A/C protein and exhibited prominent nuclear senescence as observed in aging cells. When *LMNA*^R225X/WT^ dermal fibroblast exposed to external stimuli with electrical stimulation, markedly increased in the percentage of cells with nuclear senescence as well as apoptosis were observed as compared with control dermal fibroblast. These findings suggest that *LMNA*^R225X/WT^ somatic cells have increased susceptibility to nuclear senescence and apoptosis after exposure to external physical stress. Furthermore, our pre-screening results showed that activation of stress response pathway with MEK1 contribute to increase apoptosis of *LMNA*^R225X/WT^ dermal fibroblast after electrical stimulation.

Next, we generated disease-specific iPSC lines from the proband with *LMNA*^R225X/WT^ and differentiated them together with another *LMNA* haploinsufficiency line, LMNA^Frameshift/WT^ into cardiomyocytes. Interestingly, *LMNA*^R225X/WT^ and LMNA^Frameshift/WT^ iPSC-CMs showed normal phenotypes without significant nuclear abnormalities and electrophysiological properties at baseline as control iPSC-CMs. However, when these cardiomyocytes were subjected to electrical stimulation to mimic their*in-vivo* host environment, they exhibited typical nuclear abnormalities resembling previous pathological findings from explanted human cardiomyocytes from individuals with *LMNA*-related DCM [28]. Furthermore, electrical stimulation significantly increased cellular apoptosis of *LMNA* haploinsufficiency iPSC-CMs, but not in control iPSC-CMs. The biological relevance of lamin A/C protein in cardiomyocytes was further verified by *in-vitro* knock-down of *LMNA* with adenoviral transient expression shRNA in control iPSC-CMs. Indeed, shRNA treated control iPSC-CMs mimicked the phenotypes changes as well as the susceptibility to apoptosis after electrical stimulation as *LMNA*^R225X/WT^ and LMNA^Frameshift/WT^ iPSC-CMs. More importantly, the increased susceptibility of apoptosis with electrical stimulation in the shRNA treated control- and the mutated *LMNA* iPSC-CMs could be attenuated or even completely abolished by pharmacological blockade of the MEK1/ERK1/2 pathway.

Currently, insights into the pathogenesis of *LMNA*-related DCM are mainly based on the observations from experimental animal models. In both lamin A/C-deficient (*LMNA*^−/−^)[16] and lamin A/C-insufficient (*LMNA*^+/−^)[18] mouse models, animals were born with normal functioning hearts, but eventually developed premature cardiac aging with various degree of atrioventricular block, atrial arrhythmias, DCM, and ventricular tachycardia, closely resembling to those observed in humans with heterozygous *LMNA* mutations. Although nuclear fragility secondary to lamin A/C-insufficiency has been postulated to render cardiomyocytes more susceptible to mechanical stress-induced damage and apoptosis [29], the application of mechanical stress to lamin A/C-insufficient (*LMNA*^+/−^) mice failed to enhance apoptosis or accelerate DCM.[30] On the contrary, histological studies of the hearts of both lamin A/C-insufficient (*LMNA*^+/−^), and lamin A/C-deficient (*LMNA*^−/−^) mice revealed typical nuclear abnormalities and apoptosis in cardiomyocytes. Interestingly, it appeared that electrically-active, conducting cardiomyocytes such as atrioventricular nodal cells exhibited more severe nuclear injuries and developed apoptosis much earlier than the non-conducting counterparts[18]. In concordance with these observations, previous clinical studies [7, 8] and our results showed that progressive conduction system disease is the early manifestation before the onset of DCM. In this study, family screening revealed that two of the affected subjects with *LMNA*^R225X/WT^ mutation had subclinical conduction system disease before any clinical manifestation of DCM.

Accordingly, we hypothesized that lamin-insufficiency renders the cells more susceptible to apoptosis induced by electrical stimulation. Furthermore, we tested whether activation of stress response MEK1/ERK1/2 pathway contribute to the pathogenesis of apoptosis induced by electrical stimulation in LMNA-related DCM. Notably, ERK1/2, the downstream kinase of MEK1, is a member of the mitogen-activated protein kinases super family mediating cell proliferation and apoptosis depending on the stimuli and cell types. This is a particularly attractive explanation for *LMNA*-related DCM, as cardiomyocytes are constantly exposed to an alternating electrical field whereas cells in non-cardiac tissues might remain relatively unharmed, despite exhibiting the same nuclear abnormalities. Experimental studies using adult rat ventricular cardiomyocytes had demonstrated that under rapid electrical pacing, cultured cardiomyocytes had enhanced apoptosis as well as activation of ERK1/2 [31]. In a mouse model of another laminopathy, autosomal Emery-Dreifuss muscular dysfunction due to a missense H222P *LMNA* mutationalso manifests with DCM and atrioventricular conduction abnormality. In this model, ERK activation in heart muscle has been shown to play a key role in the pathogenesis of DCM [17]. More importantly, pharmacological inhibition of ERK activation prevented the development of DCM in these *LMNA*^H222P/H222P^ mice [4]. In this study, pharmacology blockade of MEK1 pathway with U0126 and selumetinib significantly attenuated apoptosis in *LMNA*^R225X/WT^ as well as LMNA^Frameshift/WT^ iPSC-CMs after electrical stimulation, and selumetinib showed a consistent effect with a much greater efficacy (25 nM vs. 10 μM to rescue apoptosis). These findings in human iPSC-CM model provides further evidences to support the notion that pharmacological manipulation of MEK1 pathway might be a potential therapeutic target in *LMNA*-related DCM as reported in prior animal models [32]. Although selumetinib has been tested as anti-cancer therapy [33-35], its long-term safety and efficacy for treatment of *LMNA*-related DCM remain unclear.

Very recently, hyperactivation of the mammalian target of rapamycin complex 1 (mTORC1) signaling pathway has been demonstrated in mouse models of laminopathies to be contributory to the cardiac and skeletal muscle defects [36, 37]. In fact, it has been shown that the pathway can determine the choice of cell fate at least in part between senescence and quiescence [38]. More importantly, inhibition of the pathway with rapamycin, a specific mTORC1 inhibitor alleviates cardiomyopathies in these mouse models [36, 37]. In a stark contrast, the administration of rapamycin to both *LMNA*^R225X/WT^ and LMNA^Frameshift/WT^ iPSC-CMs in our study failed to attenuate the electrical-stimulation induced apoptosis. This suggests that the observed attenuation of electrical-stimulation induced apoptosis of *LMNA* iPSC-CMs from ERK1/2 inhibition is not secondary to the indirect mTORC1 inhibition at least in these two *LMNA* mutations. In fact, it is well recognized that mutations in *LMNA* cause a protean range of human diseases with a high mutation-specific tissue-selectivity. Conceivably, different pathogenic mechanisms may be involved in different mutations. For instance, Hutchinson-Gilford progeria syndrome, the classical premature aging syndrome is due to a missense *LMNA* mutation leading to partially activates a cryptic splice donor site in exon 11, thereby producing an abnormal lamin A protein, progerin. The intracellular accumulation of progerin, as a toxin peptide, results in nuclear blebbing, mitotic abnormalities, replicative senescence, and accelerated telomere shortening [39]. In fact, the potential beneficial effects of rapamycin in human laminopathies were first demonstrated in fibroblasts from patients with Hutchinson-Gilford progeria syndrome [40]. Rapamycin promoting clearance of progerin by enhancing autophagy, has been demonstrated to abolish the development of nuclear bleeding and to delay the onset of cellular senescence of Hutchinson-Gilford progeria syndrome fibroblasts [40]. At a closer look, while the two *LMNA* mutations described in our study share very similar cardiac phenotypes (conduction abnormality, hear failure, ventricular tachyarrhythmia) with the previous reported *LMNA* mutation causing autosomal Emery-Dreifuss muscular dystrophy [41] presumably due to halo-insufficiency studied in the mouse models [17. 41, 42], none of our patients have clinical evidences of skeletal muscle abnormalities as in the original human laminopathy [41] and/or the derived mouse models of cardiac laminopathies [17. 41, 42]. Noteworthy, in the mouse model of the autosomal Emery-Dreifuss muscular dystrophy (missense H222P *LMNA* mutation), impaired authophagy due to the impaired mTORC1 pathway has been demonstrated which responded to mTORC1 inhibition [36]. Unlike the *LMNA*^R225X^ mutation reported in our study, the H222P *LMNA* mutation in addition to halo-insufficiency may presumably produce and accumulate non-functioning lamin proteins similarly to progerin in Hutchinson Gilford progeria syndrome necessitating intrinsic autophagic mechanisms to clear them. However, the *LMNA*^R225X^ mutation results in a premature stop-codon, which leads to the production of a very small truncated protein, thereby the predominant pathogenic mechanisms may be related to the loss of function of lamin due to halo-insufficiency rather than toxin peptide accumulation. Consequently, the enhanced autophagy due to the mTORC1 inhibition may have little beneficial effects to our cells. Given the diversity and high mutation-specific tissue-selectivity of *LMNA* related human diseases, human iPSCs appear to be a unique platform not only allowing investigating human diseases in a human platform in a mutation-specific manner, but also facilitating preclinical drug testing.

This study has several limitations. First, due to the lack of specific markers to allow effective sorting of different subset of cardiomyocytes such as nodal, atrial and ventricular myocytes, the effects of *LMNA* mutation as well as their in-vitro response to electrical stimulation remain unclear. Second, the results of this study may not be translated to *LMNA*-related DCM due to other mutations. Nevertheless, the ability to reproduce the disease phenotypes of *LMNA*^R225X/WT^ iPSC-CMs using non-specific knock-down of *LMNA* with shRNA in control iPSC-CMs, suggesting that our results should be applicable to *LMNA*-related DCM due to haploinsufficiency.

In conclusion, we results show that patient-specific iPSC-CMs can be used to model the pathophysiology of *LMNA*-related DCM; and provide novel insight for potential therapeutic intervention for this most common form of familial DCM.

## METHODS

### Clinical history and genetic phenotype

A 56 year-old Chinese man (II-7) presented to us with atrial fibrillation and cardioembolic stroke. He had history of progressive conduction system disease, evolving from 1^st^ degree to 3^rd^ degree atrioventricular block, and required permanent cardiac pacemaker implantation at age of 49 years (Table 1, and Figure 1A). Subsequently, he developed progressive heart failure and transthoracic echocardiogram revealed dilated right and left ventricles with impaired left ventricular ejection fraction of 35%. Then he underwent upgrading of his pacemaker to cardiac resynchronization therapy with defibrillator, and had received multiple device therapy for ventricular tachyarrhythmias during follow-up. Sequencing of the *LMNA* gene revealed a heterozygous single base exchange (672G→T) in exon 4, resulting in an R225X nonsense mutation (truncation in the coil 1B domain of both lamin A and C proteins), previously known to be associated with familial DCM (Figure 1B and IC)[43-45]. Indeed, he had a strong family history of DCM, conduction system disease and sudden death spanning 3 generations (Figure 1A). His father (I-1) suffered sudden death at age of 59, and one of his sisters (II-2) suffered from atrial fibrillation, heart failure and 3^rd^ degree atrioventricular block with pacemaker implanted, who died suddenly and had documented ventricular fibrillation from her pacemaker memory. In addition, his two other sisters (II-5 and II-9) both had atrial fibrillation and 3rd degree atrioventricular block with permanent pacemaker implantation at 51 years and 48 years respectively. Subsequent screening of members of his family revealed asymptomatic 1^st^ to 2^nd^ degree atrioventricular block in 3 younger, apparently healthy family members (III-2, III-3, and III-4), in whom 24-hour ECG monitoring detected non-sustained ventricular tachyarrhythmia in two of them. Cardiac magnetic resonance imaging revealed patchy fibrosis over right ventricular myocardium (III-3 and III-4). Genetic testing showed that these family members had the same heterozygous *LMNA* mutation, confirming autosomal dominant inheritance in this family.

**Table 1:** Cardiac manifestations in affected subjects from a single extended family with *LMNA* R225X mutation

		Cardiac Manifestations (age of diagnosis in years)					
Affected subjects	Sex	AV block	AF	VT/SCD	Cardiomyopathy	AICD/Pacemaker	Age of death
I-1	M	?	?	+ (59)	?	-	(59)
II-2	F	CHB (43)	+ (46)	+ (53)	+ (43)	+ (43)	(53)
II-4	F	2° HB (53)	-	-	-	-	-
II-5	F	CHB (51)	+ (52)	-	-	+ (51)	-
II-7	M	CHB (49)	+ (49)	+ (50)	+ (51)	+ (52)	-
II-9	F	CHB (48)	+ (48)	-	-	+ (48)	-
III-2	F	2° HB, (43)	-	+ (44)	-	+ (44)	-
III-3	F	1° HB, PR: 360ms (43)	-	-	-	-	-
III-4	M	1° HB, PR: 320ms (39)	-	+ (39)	+ (39)	-	-

**Abbreviations:** 1° HB: first degree heart block; 2° HB: second degree heart block; AF: Atrial fibrillation; AICD: automatic implantable cardioverter defibrillator; AV block: atrio-ventricular block; CHB: complete heart block; PR: P-R interval; SCD: sudden cardiac death; VT: ventricular tachyarrhythmia.

### Mitogen-Activated Protein Kinase (MAPK) phosphorylation analysis

Total cell lysate obtained from mutant and control primary fibroblast underwent electric stimulation from five individual experiment was subjected to MAPK pathway phosphorylation analysis using the Milliplex MAP 10plex MAPK/SAPK signaling kit (Millipore, St. Charles, Missouri, USA) according to manufacturer instructions.[46] Bio-Plex Manager software version 4.1.1 was used for data acquisition and analysis. Each lysate was measured in duplicated wells and the results were present as mean with standard error.

### Generation of disease-specific induced pluripotent stem cells

For the generation of iPSCs, we recruited the proband (II-7) (LMNA^R225X/WT^) and another patient with frame-shift-mutated LMNA (contained a GCCA insertion at base 50 in *lamin A/C* gene, LMNA ^Frameshift/WT^)along with one healthy age- and sex-matched control subject (LMNA^WT/WT^). The male patient (45 years old) bearing framshift-mutated LMNA was chosen also suffered from DCM and conduction system defectas previously described [27]. The study protocol of procurement of human tissue for the generation of iPSCs was approved by the local Institutional Review Board and was registered at the Clinical Trial Center, the University of Hong Kong (HKCTR-725, http://www.hkclinicaltrials.com).

After obtaining Informed consents from all participants, skin biopsies were taken, minced and then plated in 6-well culture dishes with 10% FBS medium as previously described[47]. Dermal fibroblasts growing out from the skin tissues were expanded and transduced with lentiviruses encoding OCT4, SOX2, KLF4, and c-MYC. Putative iPSC clusters were typically observed 14-21 days after lentiviral transduction, which were manually dissected and expanded in new Matrigel™-coated 6-well plates (Supplementary Figure 1). The authenticity of the human iPSCs was confirmed with the expression of a panel of pluripotent markers, transgene silencing, OCT4 promoter demethylation, and teratoma formation after inoculation into severe combined immunodeficiency mice (Supplementary Figure 1 and Result section in Supplementary Appendix). All the stem cell characterization of LMNA^Frameshift/WT^ iPSC has been reported previously [27].

### *In vitro* cardiac differentiation and characterization of cardiomyocytes

To induce cardiac differentiation, undifferentiated iPSCs were co-cultured with endoderm-like cells as described [48-51]. Briefly, undifferentiated iPSCs were dissociated into clumps using dispase (Invitrogen, CA, USA) and cultured in suspension using low attachment plates to form embryoid bodies. Embryoid bodies were transferred onto irradiated endoderm-like cells for co-culture. Beating outgrowths from iPSC embryoid bodies were micro-surgically dissected 20 days after induction of cardiac differentiation, followed by enzymatic dissociation into single isolated cardiomyocytes for further experiments. Rescued effect of electrical field-induced apoptosis of iPSC-CMs was studied by using mitogen-activated protein kinase kinase 1(MEK1)-inhibitor, 10 μM U0126 (dissolved in DMSO from Toricis), or 25 nM selumetinib, AZD6244 (dissolved in DMSO from Selleck Chemicals), an anti-cancer agent at phase I-II clinical trials; or 0.68 mM rapamycin (dissolved in DMSO from Sigma),[40] a specific inhibitor of the mTORC1 pathway. Standard immunofluorescence staining of alpha-actinin and electrophysiology analysis by patch clamping were performed to confirm their cardiac phenotypes [52-54].

### shRNA mediated knockdown of LMNA

To verify the functional effect of *LMNA* in cardiomyocytes, the 18-nucleotide shRNA sequences designed to knockdown *LMNA* were tested in control iPSC-CM. shRNA against the common region of lamin A and C RNA position 609-627 from the start codon was cloned into pAd-GW/U6 vectors, and pAd-GFP was used as control. Adenovirus for shRNA against *LMNA* and control GFP vectors were produced using ViraPower Adenoviral Expression Kit (Invitrogen, CA, USA) and purified using Add-N-Pure Viral Purification kit (Applied Biological Materials Inc., Richmond, BC, Canada) according to manufacturer's instruction respectively. Viral titer was determined by AdEasy Viral titer kit (Stratagene, La Jolla, CA). A MOI of 10 was used for virus transduction. A knockdown efficiency of 30-40% of lamin A and C was observed.

### Electrical field stimulation

To mimic the microenvironment of cardiomyocytes, iPSC-CMs were subjected to electrical stimulation. Cells were seeded on 13-mm glass cover slips (Nunc A/S, Rockilde, Denmark) and mounted inside a 6-well plate filled with corresponding medium. Electrical stimulation was then delivered to the cultured cell with carbon electrodes using an eight-channel C-Pace cell culture stimulator (Ion-Optics Co., Milton, MA, USA) at 6.6 V/cm, 1 Hz, 2 ms with alternating polarity for 4 hours [31]. Total number of cells was counted using standard cytometry by two blinded individuals before and after electrical stimulation.

### Terminal deoxynucleotidyl transferase dUTP nick end labeling (TUNEL) assay for apoptosis

TUNEL assay was performed using In Situ Cell Death Detection Kit, Fluorescein (Roche Applied Sciences, Mannheim, Germany) according to manufacturer's protocol. Cells were grown on glass coverslips, and after assigned treatment, cells were fixed and permeabilized with 0.1% Triton X-100 in 0.1% sodium citrate for 2 minutes on ice. Cells were then incubated in TUNEL reaction mixture at 37°C for 60 minutes in a humidified dark chamber. Coverslips were mounted onto glycerol-based mountant. Images were acquired using Carl Zeiss fluorescence microscope with AxioVision 6.0 software (Zeiss GmbH, Gottingen, Germany).

Detection of apoptotic cells upon electrical stimulation was further quantified by the APO-BrdU TUNEL Assay Kit (Molecular Probes, Inc, Eugene, OR). The dissociated cells were fixed in 1% (w/v) paraformaldehyde on ice for 15 mins and then washed twice with DPBS at 300 × g for 5 mins. The fixed cells were stored in ice-cold 70% (v/v) ethanol at −20 ° C before nicked ends labeling reaction. Before incubation in TUNEL labeling solution for an hour at 37 ° C (10 μl reaction buffer, 0.75 μl TdT enzyme, 8 μl of BrdU and 31.25 μl dH_2_O), the cells were washed with wash buffer. After further washing by rinse buffer, the cells were stained with the Alexa Fluor^®^ 488 dye-labeled anti-BrdU antibodies for half an hour. The propidium iodide/RNase A staining buffer was finally added to the cell prior to flow cytometry analysis. The positively labeled cells (green signal), a population with DNA fragmentation, were counted as % of apoptotic cells.

### Statistical Analysis

Data are expressed as mean ± SEM. Statistic analysis was performed with the unpaired student *t*-test paired sample *t*-test as appropriate. Calculations were performed using SPSS software (version 12.0). A *P*-value < 0.05 was considered statistically significant.

## SUPPLEMENTARY DATA, TABLES AND FIGURES


