# Supplementary material for: Modeling of lamin A/C mutation premature cardiac aging using patient-specific induced pluripotent stem cells
Source: Aging (Albany NY). 2012 Dec 3;4(11):803–22. doi: 10.18632/aging.100503 (PMC3560431; doi:10.18632/aging.100503)
Supplement: Supplementary file 1 [file aging-04-803-s001.pdf]

46. Lee YK, Ng KM, Lai WH, Man C, Lieu DK, Lau CP, Tse HF, Siu CW. Ouabain facilitates cardiac differentiation of mouse embryonic stem cells through ERK1/2 pathway. Acta Pharmacol Sin. 2011;32:52-61.
47. Lai WH, Ho JC, Lee YK, Ng KM, Au KW, Chan YC, Lau CP, Tse HF, Siu CW. ROCK inhibition facilitates the generation of human-induced pluripotent stem cells in a defined, feeder-, and serum-free system. Cell Reprogram. 2010;12:641-653.
48. Mummery CL, Ward D, Passier R. Differentiation of human embryonic stem cells to cardiomyocytes by coculture with endoderm in serum-free medium. Current protocols in stem cell biology 2007;Chapter 1:Unit 1F 2.
49. Mummery C, Ward-van Oostwaard D, Doevendans P, Spijker R, van den Brink S, Hassink R, van der Heyden M, Opthof T, Pera M, de la Riviere AB, Passier R, Tertoolen L. Differentiation of human embryonic stem cells to cardiomyocytes: role of coculture with visceral endoderm-like cells. Circulation. 2003;07:2733-2740.
50. Lee YK, Ng KM, Lai WH, Chan YC, Lau YM, Lian Q, Tse HF, Siu CW. Calcium homeostasis in human induced pluripotent stem cell-derived cardiomyocytes. Stem Cell Rev. 2011;7:976-986.
51. Ng KM, Lee YK, Lai WH, Chan YC, Fung ML, Tse HF, Siu CW. Exogenous expression of human apoA-I enhances cardiac differentiation of pluripotent stem cells. PLoS One. 2011;6:e19787.
52. Lee YK, Ng KM, Chan YC, Lai WH, Au KW, Ho CY, Wong LY, Lau CP, Tse HF, Siu CW. Triiodothyronine promotes cardiac differentiation and maturation of embryonic stem cells via the classical genomic pathway. Mol Endocrinol. 2010;24:1728-1736.
53. Ng KM, Lee YK, Chan YC, Lai WH, Fung ML, Li RA, Siu CW, Tse HF. Exogenous expression of HIF-1 alpha promotes cardiac differentiation of embryonic stem cells. J Mol Cell Cardiol. 2010;48:1129-1137.
54. Au KW, Liao SY, Lee YK, Lai WH, Ng KM, Chan YC, Yip MC, Ho CY, Wu EX, Li RA, Siu CW, Tse HF. Effects of iron oxide nanoparticles on cardiac differentiation of embryonic stem cells. Biochem Biophys Res Commun. 2009;379:898-903.

SUPPLEMENTARY DATA

Supplementary Table 1. Antibodies used for immunofluorescence analysis

Usage	Antibodies	Manufacturer	Catalogue number	Dilution
**Western Blotting**	Lamin A/C (N-18)	Santa Cruz Biotechnology, CA	Sc-6215	1:500
	Beta-Actin	Santa Cruz Biotechnology, CA	Sc-47778	1:500
**IPS characterization**	OCT4	Stemgent, Cambridge, MA	09-0023	1:100
	SSEA-4		09-0006	
	Tra1-60		09-0010	
	Nanog		09-0020	
**CM staining**	Alpha-actinin	Sigma-Aldrich, St. Louis, MO	A7811	1:200
**EC staining**	Lectin	Sigma-Aldrich, St. Louis, MO	L9006	1:100
	vWF	Millipore	AB7568	1:100
**Fibroblast staining**	Fibronectin	Santa Cruz Biotechnology, CA	Sc-69777	1:100
	Vimentin		Sc-6260	1:100
**Nuclear blebbing analysis**	Lamin A/C (N-18)	Santa Cruz Biotechnology, CA	Sc-6215	1:100

Supplementary Table 2. PCR primers and conditions for reprogramming transgene silencing analysis

Gene	Accession no.	Forward/reverse (5'→3')	Annealing temperature (˚C)	Product (bp)	Cycles
Endo-OCT4	NM_001159542.1	5'- GACAACAATGAAAATCTTCAGGAGA -3'<br>5' - TTCTGGCGCCGGTTACAGAACCA -3'	57	223	30
Endo-NANOG	NM_024865.2	5'-AAGACAAGGTCCCGGTCAAG<br>5'- CCTAGTGGTCTGCTGTATTAC	57	583	30
*Exo-OCT4	NM_001159542.1	5'-TCAAGCCTCAGACAGTGGTTC3'<br>5'-GGCCCGATTCCTGGCCCTCA3'	57	236	30
*Exo-NANOG	NM_024865	5'-TCAAGCCTCAGACAGTGGTTC-3'<br>5'-CTTCAAAGCAAGGCAAGCTT-3'	57	296	30
GAPDH	NM_011406	5'- AGCCACATCGCTCAGACACC -3'<br>5'- GTACTCAGCGGCCAGCATCG -3'	60	157	30

Abbreviation: OCT4 : octamer-binding transcription factor 4 ; GAPDH: Glyceraldehyde 3-phosphate dehydrogenase.
*Forward primer was probed on EF1-alpha coding region, which is upstream of the OCT-4/NANOG cDNA sequence in the lentiviral reprogramming vector.

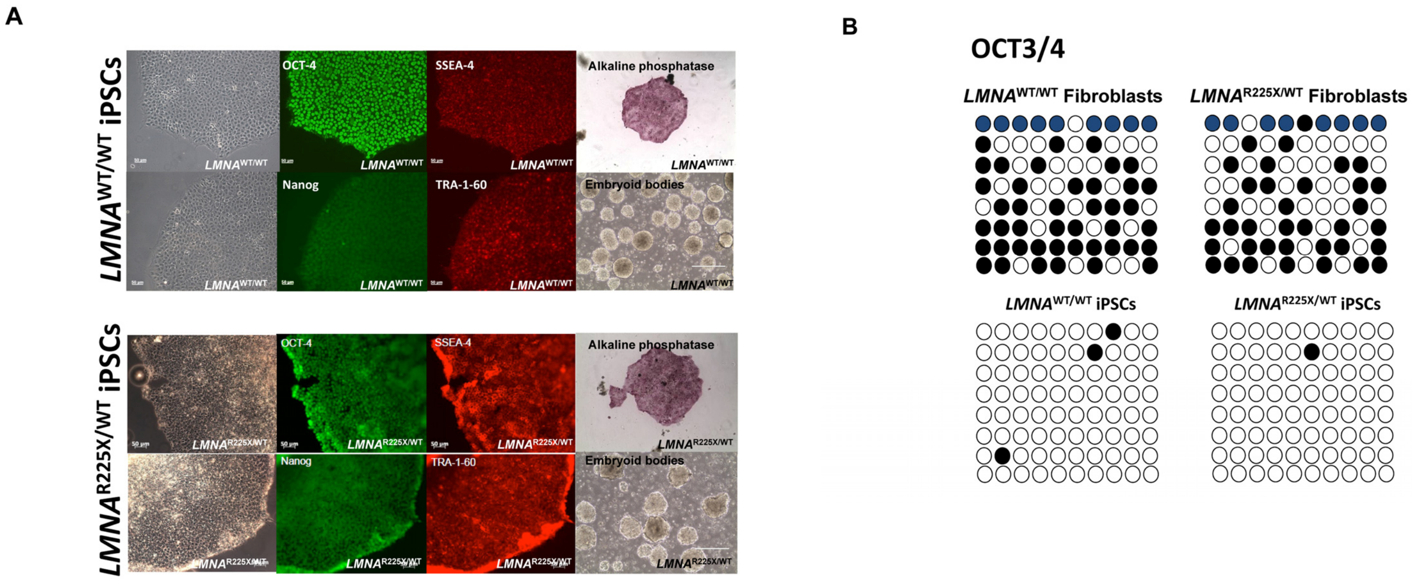

Figure 1. Generation of patient-specific iPSC lines. (A) Immunofluorescence analysis of pluripotent markers OCT4 (Green), SSEA4 (Red), NANONG (Green), and TRA-1-60; the expression of alkaline phosphatase; and embryoid body formation in representative iPSC clones derived from the proband (II:7) and the healthy control, **(B)** Oct-4 promoter methylation analysis with bisulfate pyro-sequencing in two parent fibroblast lines (LMNA$^{R225X/WT}$ and LMNA$^{WT/WT}$), and their iPSC lines.

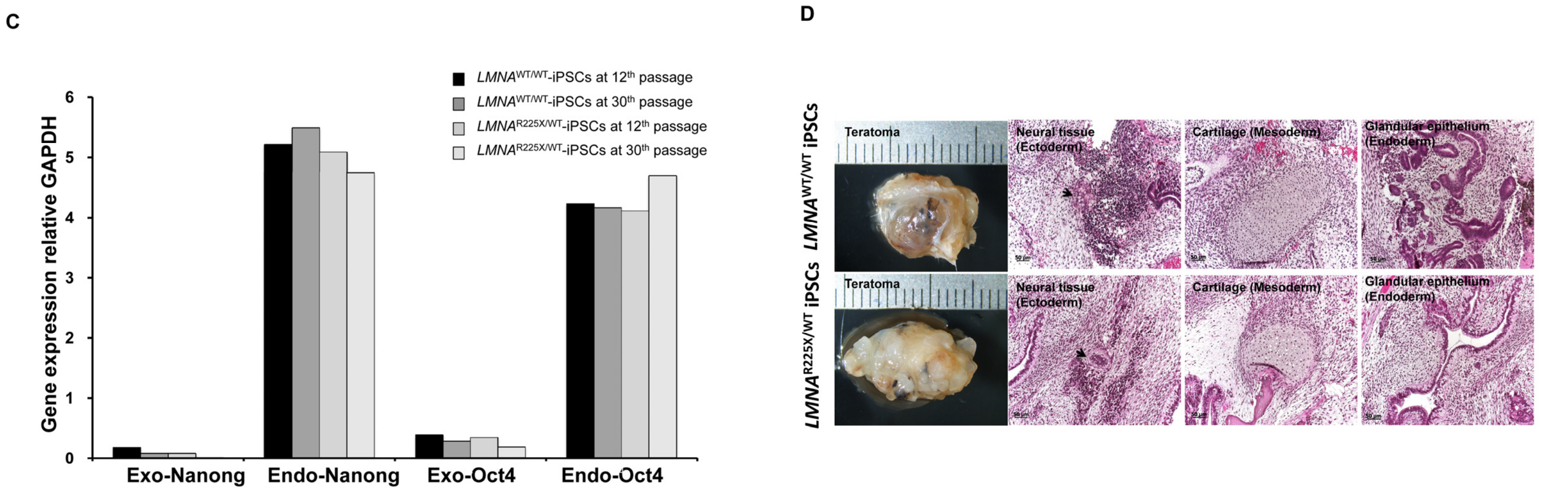

Figure 1. Generation of patient-specific iPSC lines. (C) RT-PCR analyses of the endogenous and exogenous level of OCT4 and Nanog of $LMNA^{R225X/WT}$ and $LMNA^{WT/WT}$ iPSC lines at 12th and 30th passages, **(D)** Teratoma formation and the histological section of teratoma formed 4-6 weeks after subcutaneous injection of $\textit{LMNA}^{R225X/WT}$ and $\textit{LMNA}^{WT/WT}$ iPSC lines into NOD/SCID mice.

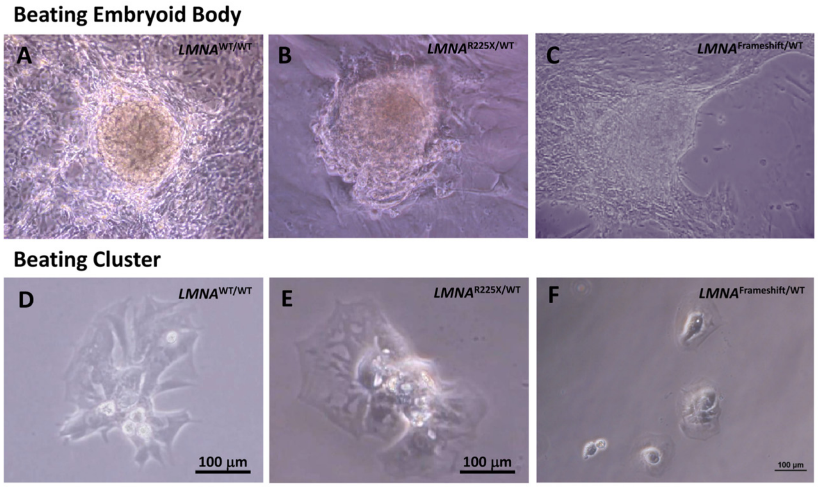

Figure 2. (A, B, & C) Beating embryoid bodies derived from $\textit{LMNA}^{R225X/WT}$, $\textit{LMNA}^{Frameshift/WT}$, and $\textit{LMNA}^{WT/WT}$ iPSCs; **(D, E, & F)** Spontaneously beating cell clusters after dissociation. Videos of these beating embryoid bodies and clusters were available in supplemental materials.

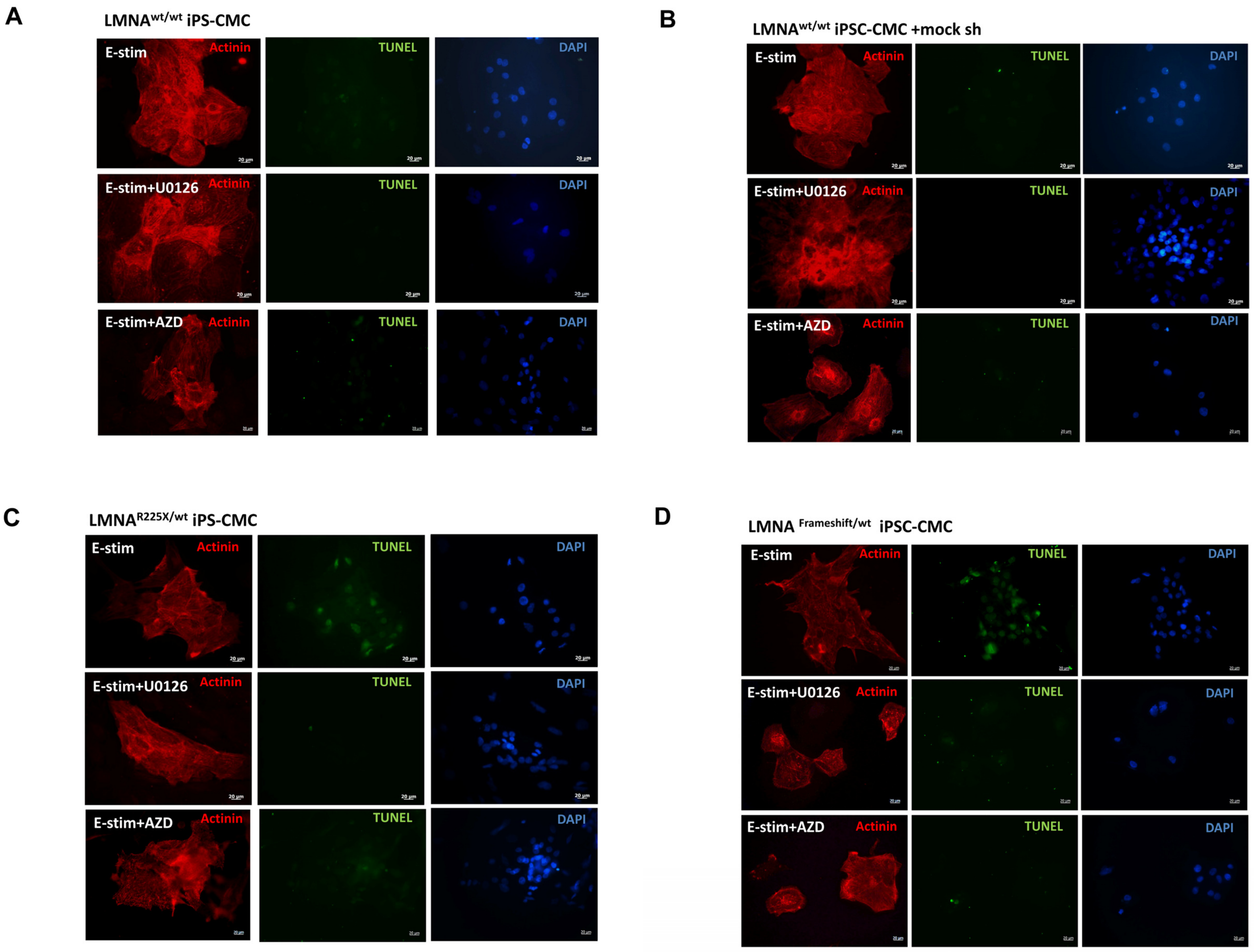

Figure 3. Electrical stimulation inducing apoptosis in cardiomyocytes derived from *LMNA*$^{R225X/WT}$ & *LMNA*$^{Frameshift/WT}$ iPSCs. Representative TUNEL assay and co-immunofluorescence staining of alpha-actinin in cardiomyocytes derived from **(A)** *LMNA*$^{WT/WT}$ iPSCs, **(B)** *LMNA*$^{R225X/WT}$ iPSCs, **(C)** *LMNA*$^{Frameshift/WT}$ iPSCs, **(D)** *LMNA*$^{WT/WT}$ iPSCs treated with mock shRNA.

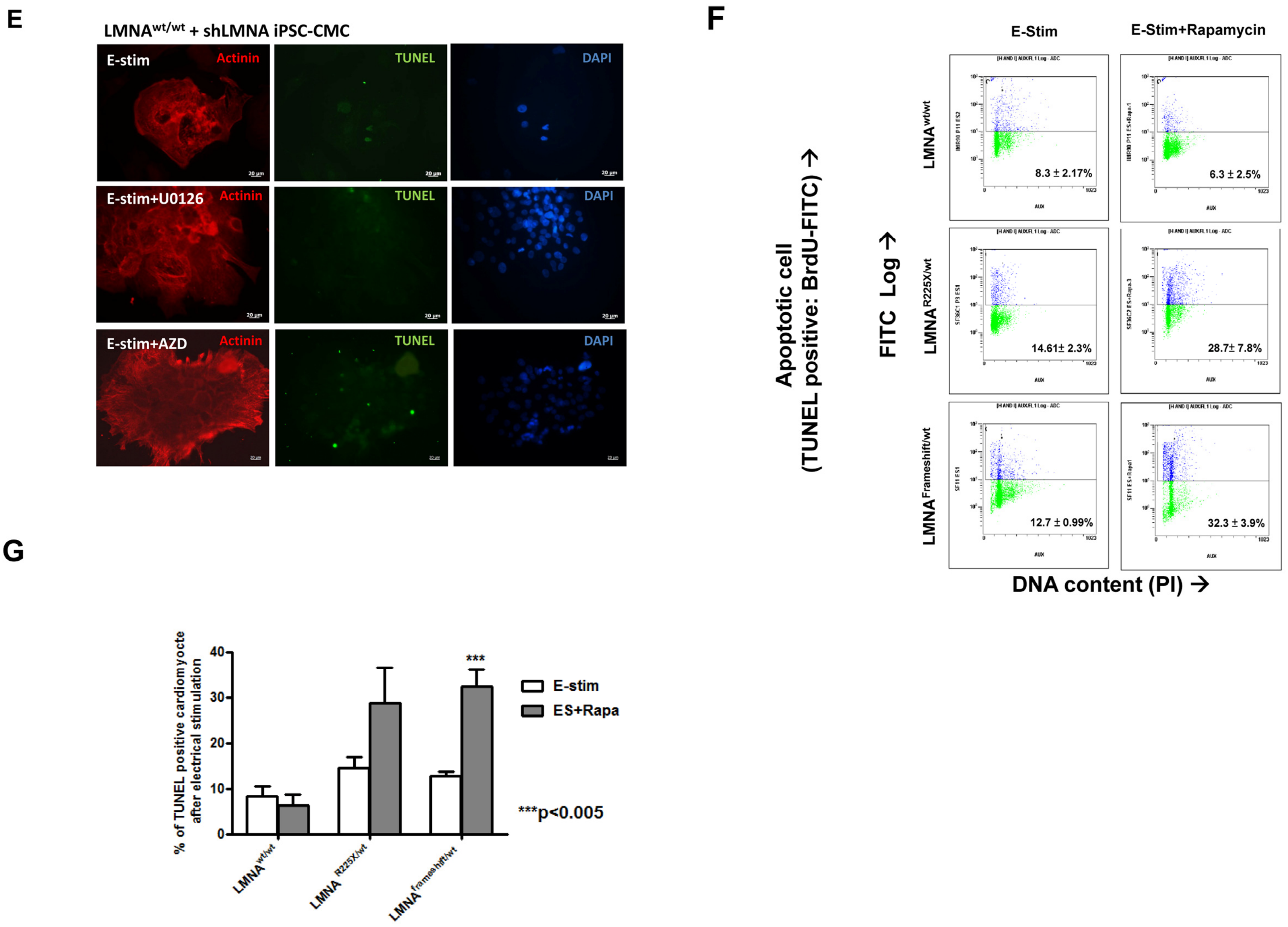

Figure 3. Electrical stimulation inducing apoptosis in cardiomyocytes derived from *LMNA*$^{R225X/WT}$ & *LMNA*$^{Frameshift/WT}$ iPSCs. **(E)** *LMNA*$^{WT/WT}$ iPSCs treated with sh*LMNA*, **(F and G)** Quantification of apoptotic cardiac differentiated iPSCs in presence of rapamycin by APO-BrdU TUNEL assay at baseline and after electrical stimulation. The percentage of cardiomyocytes with apoptosis was determined by FACS analysis by FL-1 positive gating. Unpaired t-test was performed between treatment and baseline n=3.
